# Metabolomic Changes of Human Proximal Tubular Cell Line in High Glucose Environment

**DOI:** 10.1038/s41598-019-53214-1

**Published:** 2019-11-12

**Authors:** Pascal Zhongping Wei, Winston Wing-Shing Fung, Jack Kit-Chung Ng, Ka-Bik Lai, Cathy Choi-Wan Luk, Kai Ming Chow, Philip Kam-Tao Li, Cheuk Chun Szeto

**Affiliations:** 1 Carol and Richard Yu Peritoneal Dialysis Research Centre, Department of Medicine & Therapeutics, Prince of Wales Hospital, The Chinese University of Hong Kong, Shatin, Hong Kong SAR China; 2 Li Ka Shing Institute of Health Sciences (LiHS), Faculty of Medicine, both from The Chinese University of Hong Kong, Shatin, Hong Kong SAR China

**Keywords:** Diabetes complications, Chronic kidney disease

## Abstract

Hyperglycemia causes mitochondrial damage renal tubular cells, which contribute to the progression of diabetic kidney disease. However, the metabolic aberration of renal tubular cells in an hyperglycemic milieu has not been fully elucidated. In this study, human proximal renal tubular cell line (HK-2 cell) are incubated in glucose and mannitol at 5 mM or 25 mM. Cellular metabolome was determined by capillary electrophoresis time of flight mass spectrometer (CE-TOF/MS) and capillary electrophoresis-triple quadrupole mass spectrometry (CE-QqQMS). A total of 116 metabolites were quantified. Principal component analysis (PCA) revealed excellent clustering of metabolomic changes for different treatment conditions, and exposure to glucose at 5 and 25 mM lead to distinct metabolomic profiles as compared to samples treated with serum-free medium or mannitol as osmotic control. Hierarchical clustering analysis showed a number of characteristic changes in metabolic profile following exposure to 5 mM or 25 mM glucose. Notably, lactate-to-pyruvate ratio was significantly increased, while cellular levels of citric acid, α-ketoglutaric acid (i.e. 2-oxoglutaric acid), and fumaric acid were significantly reduced after exposure to glucose at 25 mM but not 5 mM. Moreover, cellular levels of reduced glutathione and total glutathione were significantly decreased, and S-adenosylmethionine (SAM) to S-adenosylhomocysteine (SAH) ratio was significantly increased after exposure to glucose 25 mM but not 5 mM. We conclude that in response to high glucose, HK-2 cells characteristic metabolomic changes, including increase in lactate-to-pyruvate ratio, reduction in Krebs cycle metabolites, reduction in glutathione antioxidant activity, and increase in cellular methylation potential. Our results may shed light on the pathogenesis of diabetic kidney disease, but the expression of glucose metabolism-related protein and enzyme activity in HK-2 cells after hyperglycemia condition need to be confirmed by further studies.

## Introduction

Diabetes mellitus (DM) is characterized by an elevated plasma glucose concentration. Persistent hyperglycemia causes generalized vasculopathy and multi-organ damage^1–4^. Over the past decades, the prevalence of diabetes increased worldwide because of the increasing prevalence of obesity, western diet, and aging. In 2017, there were 451 million diabetic adults worldwide, and the number is expected to be 693 million by 2045^5^. Amongst all diabetic complications, diabetic kidney disease (DKD) attracts particular concern. DKD is now the cause of dialysis-dependent renal failure for over 50% of patients^6–8^.

The pathogenesis of DKD is complex and not completely understood^9,10^. High plasma glucose triggers various metabolic and cellular dysfunctions. Although glomerular pathology and podocyte injury are often considered to be the primary target of diabetic injury^11^, tubulointerstitial injury play an important part in the progression of DKD^12^. Since proximal renal tubular cells have a high mitochondrial content, they are particularly susceptible to metabolic and mitochondrial injury from hyperglycemia. Recent evidence suggests that damaged mitochondria in the renal tubular cells are an important cause of progressive tubulointerstitial damage in DKD^13^. For example, our previous study showed that urinary supernatant mitochondrial nucleic acid fragment level is elevated in DKD, and the degree correlates with the reduction in renal tubular cell mitochondrial content^14^. However, the metabolic changes of renal tubular cells in response to hyperglycemia remain incompletely elucidated. In this study, we determined the full metabolomic profile of HK-2 cells in response to hyperglycemia by capillary electrophoresis time of flight mass spectrometer (CE-TOF/MS) and capillary electrophoresis-triple quadrupole mass spectrometry (CE-QqQMS).

## Results

### Metabolomic profiling in HK-2 cell

Sample information and cell numbers are summarized in Supplementary Table 1. A total of 116 metabolites (54 and 62 metabolites in the cation and anion mode, respectively) were detected. These metabolites were involved in glycolysis, pentose phosphate pathway, TCA cycle, urea cycle, and polyamine, creatine, purine, glutathione, nicotinamide, choline, and amino acid metabolisms.

### Principal component analysis

**Figure 1:**
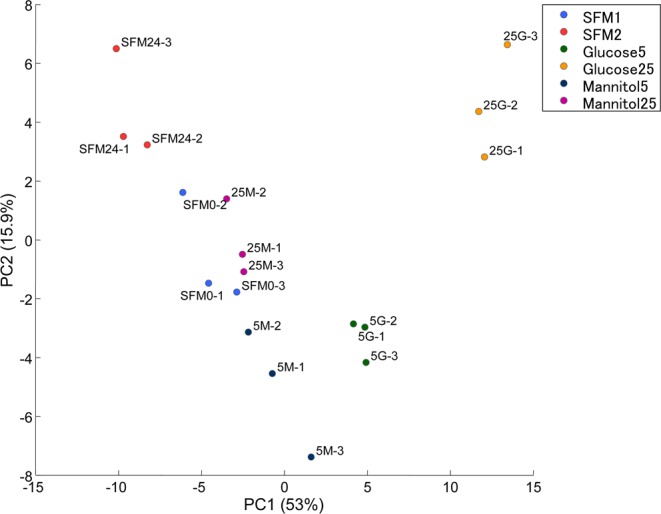
Principal component 1 (PC1) versus principal component 2 (PC2) plot based on the result of principal component analysis. (Key: SFM1, serum free medium at 0 hour; SFM2, serum free medium incubated for 24 hours; glucose5, glucose at 5 mM; glucose25, glucose at 25 mM; mannitol5, mannitol at 5 mM; mannitol25, mannitol at 25 mM. Percentage in the axis indicates the fraction of variance explained by the principal component).

Principal component analysis (PCA) was performed to compare the overall metabolomics profiles (Fig. 1; Supplementary Tables 2 and 3). In essence, PCA showed excellent clustering of metabolomic changes for different treatment conditions, and exposure to glucose at 5 and 25 mM lead to distinct metabolomic profiles as compared to samples treated with serum-free medium or mannitol as osmotic control.

### Hierarchical cluster analysis

**Figure 2:**
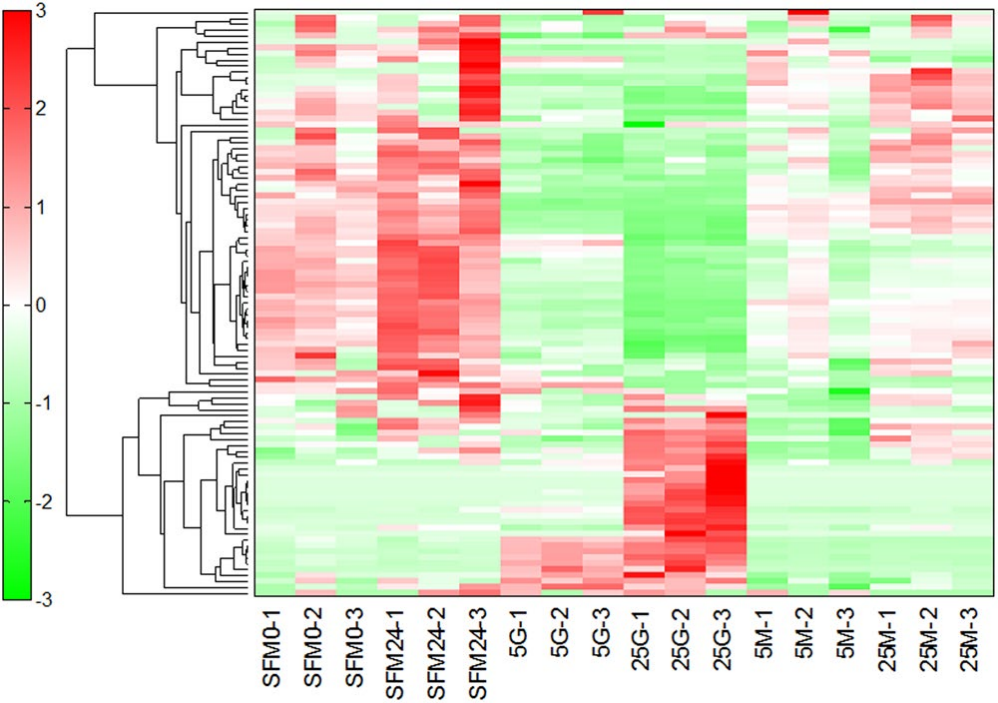
Heat map representation of metabolome profiles analyzed by hierarchical clustering analysis. (Key: SFM0, serum free medium at 0 hour; SFM24, serum free medium incubated for 24 hours; 5 G, glucose at 5 mM; 25 G, glucose at 25 mM; 5 M, mannitol at 5 mM; 25 M, mannitol at 25 mM).

Hierarchical clustering analysis was then performed to study the metabolomics profiles between various treatment conditions. The heat map representation of metabolome profiles analyzed by hierarchical clustering analysis is summarized in Fig. 2 (with complete data in Supplementary Table 4). In this analysis, a number of characteristic changes in metabolic profile are noted in HK-2 cells following exposure to 5 mM and 25 mM glucose. The level of several metabolites in HK-2 cells are specifically increased only after exposure to 25 mM glucose, but not 5 mM, including hypoxanthine, xylose 5-phosphate, inosine, guanine, ribose 5-phosphate, ribose 1-phosphate, dihydroxyacetone phosphate, ribulose 5-phosphate, fructose 1,6-diphosphate, fructose 1-phosphate, S-adenosylmethionine, and glyceraldehyde 3-phosphate (see Supplementary Table 4). Their levels were significantly higher when exposed to 25 mM glucose as compared to 5 mM glucose or serum-free medium controls by post hoc analysis with unpaired Student’s t test (details not shown). The concentrations of target metabolites, superimposed on the possible metabolite pathway map according to the hyperglycemia condition, are summarized in Supplementary Fig. 1.

### Concentration of specific metabolites

**Figure 3:**
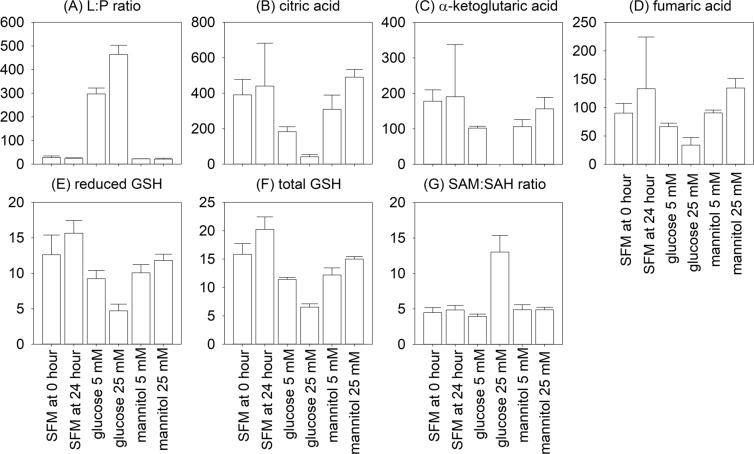
Specific metabolic changes in HK-2 cells in response to glucose. (**A**) lactate-to-pyruvate (L:P) ratio; (**B**) citric acid; (**C**) α-ketoglutaric acid; (**D**) fumaric acid; (**E)** reduced glutathione (GSH); (**F**) total GSH; and (**G**) S-adenosylmethionine (SAM) to S-adenosylhomocysteine (SAH) ratio. The unit of citric acid, α-ketoglutaric acid, and fumaric acid are pmol/10^6^ cells; The unit of reduced GSH and total GSH are nmol/10^6^ cells. Bar plots with error bars denoting standard error of mean. (SFM, serum free medium).

The absolute concentration of detectable metabolites were normalized by cell numbers, and the results are summarized in Supplementary Table 5. From the result of hierarchical clustering analysis and metabolite concentration measurement, a number of characteristic metabolic changes are noted in HK-2 cells following exposure to glucose (Fig. 3). Specifically, lactate-to-pyruvate ratio was 25.2 ± 1.8, 297.0 ± 25.1, and 464.5 ± 38.6 for HK-2 cells exposed to serum-free medium, glucose 5 and 25 mM, respectively (Student’s t test, p = 0.003 for both comparisons) (Fig. 3A). Cellular levels of citric acid, α-ketoglutaric acid, and fumaric acid were substantially reduced after exposure to 25 mM (but not 5 mM) glucose as compared to serum-free medium (Student’s t test, 42.0 ± 12.5 vs. 440.8 ± 241.5, 0.0 ± 0.0 vs. 190.5 ± 147.3, and 33.8 ± 13.3 vs. 133.3 ± 91.2 pmol per 10^6^ cells, respectively; p = 0.001 for all comparisons) (Fig. 3B–D). Similarly, both reduced glutathione (GSH) and total glutathione levels were substantially decreased after exposure to 25 mM (but not 5 mM) glucose as compared to serum-free medium (Student’s t test, 4.71 ± 9.34 vs. 15.65 ± 1.77, and 6.54 ± 5.82 vs. 20.21 ± 2.23 nmol per10^6^ cells, p = 0.002 and 0.006, respectively) (Fig. 3E,F). In contrast, there was a significant increase in S-adenosylmethionine (SAM) to S-adenosylhomocysteine (SAH) ratio after exposure to 25 mM (but not 5 mM) glucose as compared to serum-free medium (Student’s t test, 13.01 ± 2.35 vs. 4.85 ± 0.63 pmol per 10^6^ cells, p = 0.02) (Fig. 3G), indicating an increase in cellular methylation potential.

## Discussion

Mitochondrial damage and dysfunction are common in a hyperglycemic environment. Although podocyte and glomerular mesangial cells are usually considered as the cardinal cell types affected by diabetic kidney disease, renal tubular cells have a high mitochondrial content and may be specifically affected by hyperglycemia. In this study we determine the full metabolomic profile of HK-2 cells in response to hyperglycemia and compare the metabolite levels between HK-2 cells stimulated with high glucose, osmotic control, and serum-free medium. In our study, a total of 116 metabolites were detected based on the HMT metabolite database, which encompass most of the pathways involved in cellular metabolism of small molecules. Scatter plots of PCA of overall metabolomics profiles indicated that the high glucose group does not overlap with any other group, indicating that HK-2 cells have a distinct metabolomic profile in response to high glucose exposure. The metabolic response of HK-2 cell has been reported previously^15,16^. A recent study used metabolomic platform to explore the detailed metabolites change in diabetic mouse in response to high glucose^17^. However, comprehensive metabolomics analysis in HK-2 cell in response to hyperglycemia is lacking, and is the main focus of our present study.

Clinical biomarkers for the diagnosis and monitoring of diabetic kidney disease are much needed. Traditional markers (for example, urinary albumin excretion rate and kidney function) have modest accuracy. The application of metabolomic technologies provides a hypothesis-free method for the identification of valuable biomarkers, and has been explored for the identification of biomarkers so as to facilitate early diagnosis of diabetic kidney disease. For example, Looker *et al*.^18^ used a mass spectrometry method and noted that the arginine methylated derivatives of protein turnover are predictive of rapid renal function loss in DKD. On the other hand, Darshi *et al*.^19^ noted that mitochondrial function, particularly the fatty acid oxidation pathway, plays a pivotal role in the pathogenesis of DKD. In contrast, Zhang *et al*.^20^ reviewed 12 studies published before March 2015, and note that products of lipid and amino acids metabolisms were frequently affected in DKD. More importantly, there were substantial differences in the results between individual metabolomic studies, which may be related to differences in patient population and selection, as well as the technique being used^20^. Taken together, available data in this area are fragmented, and our present study contribute to the understanding of this difficult subject.

Hierarchical clustering analysis and detailed metabolite level measurement showed that the high glucose group had increased lactate-to-pyruvate ratio, reduction in TCA cycle metabolites and anti-oxidative capacity (as represented by the cellular glutathione levels), and increase in cellular methylation potential (as represented by the elevated SAM-to-SAH level). Our result is in line with previous reports on failing myocardium, which is found to show reduction in fatty acid oxidation and respiratory chain activity, as well as increased glycolysis and glucose oxidation^21^. Although proximal tubular cells are highly oxygen demanding under physiological condition, it is logical to expect that when HK-2 cells have sufficient glucose in their environment^22,23^, they use the glycolytic pathway for energy supply. Glucose is convert to lactate, resulting in the high lactate-to-pyruvate ratio. Since the cells have sufficient energy from glycolysis, TCA cycle is turned off, resulting in the reduction in TCA metabolites, including citric acid, α-ketoglutaric acid, and fumaric acid. In this regard, Sas *et al*.^24^ also reported that *de novo* synthesis of fatty acids is altered in the setting of diabetes. Since oxygen is not consumed for the TCA cycle, the cell would have an increased in demand on their anti-oxidative machinery, e.g. glutathione, for relieving the oxidative stress.

A number of limitations of our present study should be noted. Although all cells were washed before harvesting and the metabolomic profile was not directly interfered by the ingredient of the culture medium, we did not measure substrate utilization from the medium, which is equally important and interesting as the metabolome products. Second, glucose at 25 mM that we used is higher than the plasma glucose concentration in most diabetic patients, and the metabolic changes that we observed may represent an extreme condition. The time and dose-dependent glucose transporters expression has not been determined. The sample size was small and confined to one cell type. The HK-2 cells that we used could not fully mimic the situation of proximal tubular cell in the setting of diabetes. Specifically, HK-2 cells are immortalized kidney-derived cell lines, which do not entirely resemble their native counterparts. Further studies on primary culture of renal parenchymal cells would be desirable. Other renal cell lines may also be used for validation. Specifically, podocytes is responsible for the synthesis of extracellular matrix of the glomerular basement membrane, maintenance of glomerular capillary integrity, and regulation of the glomerular capillary barrier, but our result on renal tubular cells could not be extrapolated to podocytes. On the other hand, mesangial cells play an important role in the accumulation of extracellular matrix in nodular glomeruloscerlosis of diabetic kidneys^25^, while glomerular endothelial cell protein-restrictive barrier function is altered in a high glucose environment^26^. Second, diabetic kidney disease is a chronic process. Moreover, the metabolomic profiles changes secondary to hyperglycemia that we demonstrate may not be unique to the kidney, and control cell lines from other organ systems would be needed. It is important to note that our study does not aim to determine the molecular mechanism of glucose-induced renal tubular cell injury. Based on the current literature, cell injury, inflammation, oxidative stress, and defective mitophagy are responsible for the diabetes-related kidney dysfunction. The process has been reviewed in detail recently^13,27^. Specifically, the role of the polyol pathway in diabetic kidney disease and the therapeutic value of specific aldose reductase inhibitors are undergoing active investigations^28,29^. Our result also provides direct support to the current notion that transient hyperglycemia leads to long term epigenetic changes, such as DNA and histone methylation, which probably mediates the persistent alteration in gene expression despite subsequent satisfactory glycemic control^30^. Taken together, our result should be considered preliminary. Further study must focus on intact animals or clinical specimens from patients, which should have a better representation of the diabetic milieu.

In addition to validation, our result suggests that other experiments would further our understanding on the pathophysiology of diabetic kidney disease. For example, short term hyperglycemia may not reveal the long-term metabolic alterations in kidney cells. Prolonged exposure to hyperglycemia may be considered (e.g. by cell culture in high glucose for several passages). Specific inhibitors of glucose transporter may be added to see if it can reverse the metabolomic profiles changes.

## Materials and Methods

### Materials and chemicals

In this series of experiments, HK-2 cells were purchased from American Type Culture Collection (ATCC® CRL-2190™, Manassas, VA, USA). Corning® Cell Culture Flasks were used (Sigma-Aldrich Corp, St Louis, MO, USA). Keratinocyte Serum Free Medium (K-SFM) supplemented with bovine pituitary extract (BPE) and epidermal growth factor (EGF), Penicillin G/Streptomycin, and 0.05% (w/v) Trypsin-0.53 mM EDTA solution were purchased from Thermo Fisher Scientific (Waltham, MA, USA). All other study chemicals were purchased from Sigma-Aldrich.

### HK-2 cell culture

HK-2 cell was cultured in 6-well plate with glucose-free K-SFM, supplemented with 0.05 mg/ml BPE, 5 ng/ml EGF, 100 U/ml penicillin G and 100 mg/ml streptomycin at 37 °C in 5% CO_2_. When the cells were around 80% confluent, they were stimulated by glucose at 5 mM (low glucose) or 25 mM (high glucose) in complete medium for 24 hours, or mannitol at 5 mM and 25 mM as osmotic control. Cells were harvested at 24 hours. The experiment was performed in triplicate, with one extra well each for cell counting in order to normalize the metabolite concentration.

### Cell counting

The protocol of cell counting has been previously published^31^. Briefly, after each cell culture and incubation experiment, medium was aspirated and discarded. Cells were harvested by trypsin digestion, centrifuged, re-suspended, stained by 0.4% trypan blue, and then counted manually by the hemocytometer. Cell viability was confirmed by direct morphologic assessment and trypan blue exclusion assay. All wells are manually counted to ensure a similar number of cells before harvesting for metabolomic study.

### Metabolite extraction

Metabolite extraction buffer and internal standard solutions were purchased from HMT (Human Metabolome Technologies Inc., Yamagata, Japan), and the extraction protocol was provided by HMT. Briefly, internal standard solution 1 provided by HMT was freshly diluted at each experiment according to the company’s manufacturer. Mannitol 5% (w/w) in Milli-Q water was prepared and autoclaved as washing buffer. The centrifugal filter units were prewashed by 250 μL of Milli-Q water. After each cell culture and incubation experiment, medium was aspirated and discarded. The cells were then washed, followed by content extraction according to the manufacturer’s instruction. The extracted solution was then centrifuged at 2300 *g* 4 °C for 5 minute. The supernatant was further transferred to a pre-washed centrifugal filter units and centrifuged again at 9100 *g* 4 °C for 5 hour. The sample tubes were then kept at −80 °C until analysis.

### Metabolite analysis

Targeted quantitative analysis of metabolites was performed by HMT (Human Metabolome Technologies Inc., Yamagata, Japan), with the methods capillary electrophoresis time of flight mass spectrometer (CE-TOF/MS) and capillary electrophoresis-triple quadrupole mass spectrometry (CE-QqQMS). The methods of metabolite analysis have been described previously^32^. Briefly, metabolic extracts were prepared from 2–5 × 10^6^ cells with methanol containing Internal Standard Solution (HMT, Inc.). Culture medium was removed from the dish, and cells were washed twice in 5% mannitol solution (10 ml first and then 2 ml). Cells were then treated with 800 μl of methanol and 550 μl of Milli-Q water containing internal standards (H3304–1002, HMT, Inc.). The metabolite extract was transferred into a microfuge tube and centrifuged at 2300 × g and 4 °C for 5 min. Next, the upper aqueous layer was centrifugally filtered through a Millipore 5-kDa cutoff filter at 9100 × g and 4 °C for 120 min to remove proteins. The filtrate was centrifugally concentrated and re-suspended in 50 μl of Milli-Q water for analysis. Cationic compounds were measured in the cation mode of metabolome analysis using Agilent CE-TOF/MS system (Agilent Technologies, Santa Clara, CA) as previously described^33–35^. Anionic compounds were measured in the positive or negative mode of metabolome analysis using Agilent CE system and Agilent 6460 TripleQuad LC/MS system (Agilent Technologies) as previously described^33–35^.

### Data processing

Peaks detected in the CE-TOF/MS analysis were extracted using automatic integration software (MasterHands version 2.17.1.11, Keio University)^36^; peaks detected in the CE-QqQMS analysis were extracted by the MassHunter Quantitative Analysis B.06.00 service pack (Agilent Technologies). Peak information being analyzed included mass-to-charge ratio (m/z), migration time (MT), and peak area. Putative metabolites were assigned from the manufacturer’s metabolite database (Human Metabolome Technologies Inc.) on the basis of m/z and MT. The tolerance range for the peak annotation was configured at ±0.5 min for MT and ±10 ppm for m/z. The concentrations of metabolites were calculated by normalizing the peak area of each metabolite with respect to the area of the internal standard and by using standard curves, which were obtained by three-point calibrations^32^. Metabolites detected were plotted on metabolic pathway maps using Visualization and Analysis of Networks containing Experimental Data (VANTED) software^37^. The pathway map in VANTED was prepared based on the metabolic pathways that are known to exist in human cells according to the information in Kyoto Encyclopedia of Genes and Genomes (KEGG) database (http://www.genome.jp/kegg/).

### Statistical analysis

Hierarchical cluster analysis (HCA) and principal component analysis (PCA) were performed by the in-house statistical software developed by the Human Metabolome Technologies Inc. Post hoc analysis to compare specific groups were performed by unpaired Student’s t test or one way analysis of variance (ANOVA) as appropriate after correction for multiple comparison. This part of statistical analysis was performed by SPSS for Windows software version 18.0 (SPSS Inc., Chicago, IL, USA). Results were expressed as mean ± SD unless otherwise specified. A P value below 0.05 was considered statistically significant. All probabilities were two-tailed.

## Conclusions

In response to high glucose, HK-2 cells characteristic metabolomic changes, including increase in lactate-to-pyruvate ratio, reduction in Krebs cycle metabolites, reduction in glutathione antioxidant activity, and increase in cellular methylation potential. Our results may shed light on the pathogenesis of diabetic kidney disease, but the expression of glucose metabolism-related protein and enzyme activity in HK-2 cells after hyperglycemia condition need to be confirmed by further studies.

## Supplementary information

Supplementary figure 1

Supplementary table 1

Supplementary table 2

Supplementary table 3

Supplementary table 4

Supplementary table 5
