# Author Correction: Metabolomic Changes of Human Proximal Tubular Cell Line in High Glucose Environment

**DOI:** 10.1038/s41598-020-57576-9

**Published:** 2020-01-29

**Authors:** Pascal Zhongping Wei, Winston Wing-Shing Fung, Jack Kit-Chung Ng, Ka-Bik Lai, Cathy Choi-Wan Luk, Kai Ming Chow, Philip Kam-Tao Li, Cheuk Chun Szeto

**Affiliations:** 1 Carol and Richard Yu Peritoneal Dialysis Research Centre, Department of Medicine & Therapeutics, Prince of Wales Hospital, The Chinese University of Hong Kong, Shatin, Hong Kong SAR China; 2 Li Ka Shing Institute of Health Sciences (LiHS), Faculty of Medicine, both from The Chinese University of Hong Kong, Shatin, Hong Kong SAR China

Correction to: *Scientific Reports* 10.1038/s41598-019-53214-1, published online 12 November 2019

The Authors missed out a previous study on a similar topic. The additional reference is listed below as Reference 1, and should appear in the text as below.

In the Discussion section,

“In contrast, Zhang *et al*.^20^ reviewed 12 studies published before March 2015, and note that products of lipid and amino acids metabolisms were frequently affected in DKD. More importantly, there were substantial differences in the results between individual metabolomic studies, which may be related to differences in patient population and selection, as well as the technique being used^20^. Taken together, available data in this area are fragmented, and our present study contribute to the understanding of this difficult subject.”

should read:

“In contrast, Zhang *et al*.^20^ reviewed 12 studies published before March 2015, and note that products of lipid and amino acids metabolisms were frequently affected in DKD. More recently, Bernardo-Bermejo *et al*.^1^ used untargeted liquid chromatography-mass spectrometry to study the metabolomic changes of HK-2 cells induced by hyperglycemia and revealed substantial alterations in the concentration of several metabolites, including hippuric acid, sorbitol, N-steaoryl valine, pyridoxine, 5’-methylthioadenosine, phenylacetylglycine, and pyroglutamic acid, which are not included in the panel of metabolite in our own study. On the other hand, we examined numerous metabolites in the Krebs cycle, amino acids synthesis, pentose phosphate pathway, glutathione synthesis, and DNA methylation machinery, which were not explored in the study of Bernardo-Bermejo *et al*.^1^ More importantly, there were substantial differences in the results between individual metabolomic studies, which may be related to differences in patient population and selection, as well as the technique being used. Taken together, available data in this area are fragmented, and our present study contributes to the understanding of this difficult subject.”
