# Supplementary material for: Metabolomic Changes of Human Proximal Tubular Cell Line in High Glucose Environment
Source: Sci Rep. 2019 Nov 12;9:16617. doi: 10.1038/s41598-019-53214-1 (PMC6851361; doi:10.1038/s41598-019-53214-1)

**Metabolomic Changes of Human Proximal Tubular Cell Line in High Glucose Environment**

Wei PZ, Fung WW, Ng JC, Lai KB, Luk CC, Chow KM, Li PK, Szeto CC.

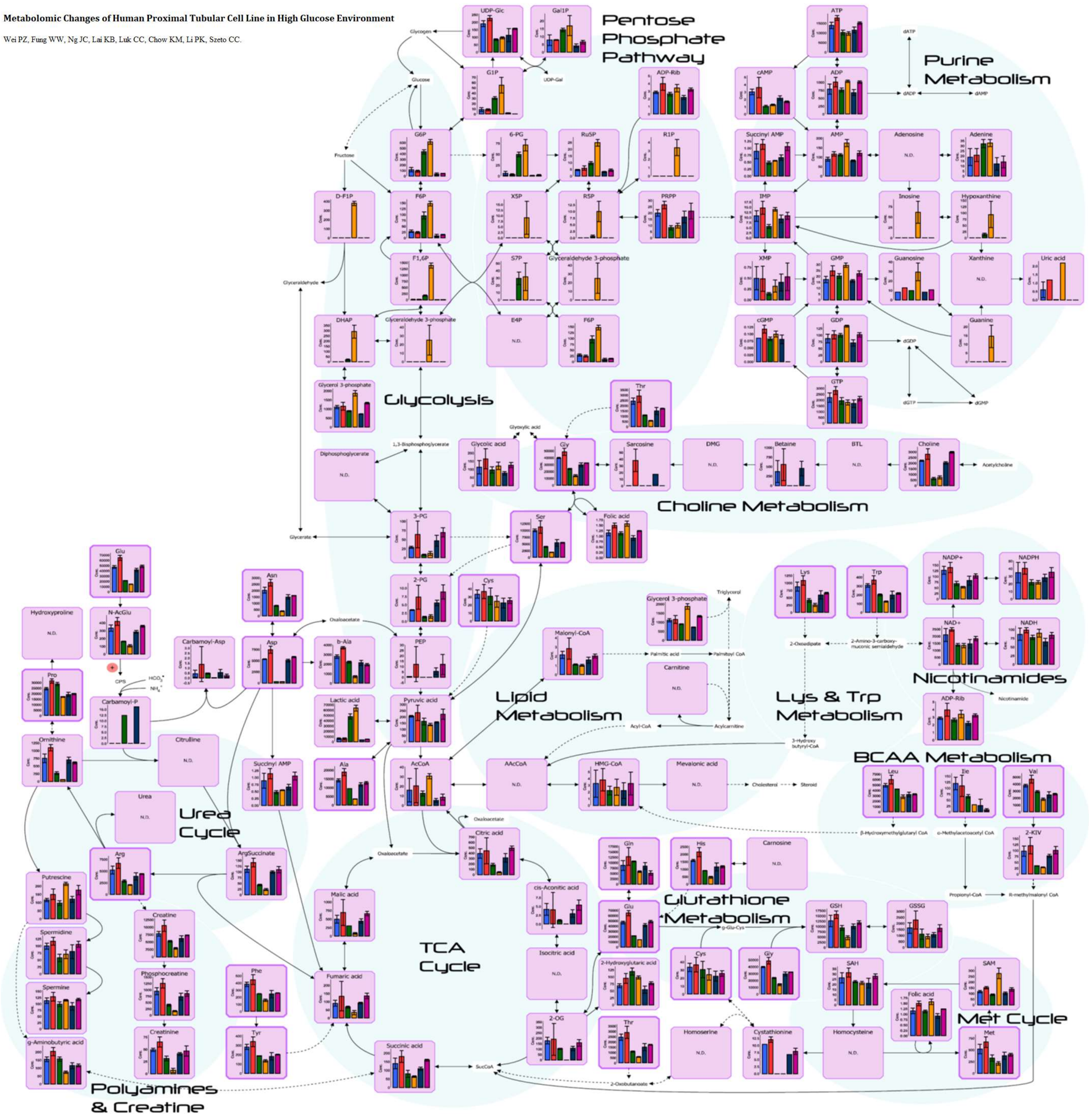

Supplement: Supplementary file 1 — Supplementary figure 1 [file 41598_2019_53214_MOESM1_ESM.pdf]
